# Book Reviews

**Published:** 1901-02

**Authors:** 


Physicians' Manual of Therapeutics. Referring Especially to the Products of the Pharmaceutical and Biological Laboratories of Parke, Davis & Co. Flexible morocco. Duodecimo, pp. 526. Detroit. 1900.

We are more than glad to present a notice of this small volume in these pages, because, among other reasons, it is a book that cannot fail to be useful to every practising physician. Moreover, it contains much valuable information relating to drugs and pharmaceutical products not found elsewhere, at least not available in such convenient form.

It is a matter of common remark, not only by the laity, but among intelligent doctors that homeopathy has compelled physicians to adopt more palatable methods in the administration of remedies. Whatever moiety of truth this may contain, we are of the opinion that the chemical and scientific pharmaceutical laboratories are chiefly responsible for improvements in this respect.

This book represents great labor, much expense, and vast knowledge of the needs of medical practitioners. A brief description of the work is merited.

Part I. is entitled, Therapeutic Suggestions, and, as its title indicates, comprises various useful hints on the prophylaxis and treatment of diseases, the names of which appear in alphabetical order, as subheads. A number of useful tables have been arranged to assist the inquirer in the differential diagnosis of the exanthemata, the calculation of metric values, thermometric equivalents, and the like. The body of the book is given over to the department of materia medica, which is, in brief, an alphabetic catalogue of drugs and seems to be thoroughly complete and abreast of the period. No secret preparations are mentioned, nor is mention made of antiquated or obsolete remedies. The various preparations of the different drugs, such as pills, tablets, capsules and elixirs, are alphabetically listed under their respective heads and may be found without confusion or delay.

It is well printed in clear faced type on thin, strong paper, with rounded corners, and is bound in flexible leather. While the nominal price of the book is $1.00, we are informed that any subscriber to the JOURNAL will receive a copy without charge and prepaid, upon request addressed to the publishers.

TRANSACTIONS OF THE MEDICAL SOCIETY OF THE STATE OF NEW YORK. For the Year 1900. Ninety-fourth annual session, held at Albany, January 30, 31 and February 1, 1900. Edited by FREDERIC C. CURTIS, M. D., Secretary. Philadelphia: Wm. J. Dornan, Printer.

As usual this society sends out its volume of transactions freighted with material that is of great scientific and general value, to every physician who is loyal to the best interests of the profession of medicine in the State of New York. An important part of the record are the reports of the standing committees. These are often overlooked by the reader, yet they contain much that deserves careful examination. Especially is this true of the reports of the committee on hygiene and of the committee on legislation. Probably it will surprise most persons to learn that 265 bills were introduced last year in the legislature affecting medical interests, either in general or in particular. It requires great activity on the part of the legislative committee to watch the progress of these bills, to promote them when useful, and to defeat them when pernicious.

The plan of publishing the proceedings of a state medical society in book form each year is a good one, and we hope to see it maintained in this body. It is a great convenience to find the whole record of work between the two covers of a single volume. The medical journals publish promptly elaborate abstracts of medical society work, so that it can be examined within a week or two by everyone interested. Then, later, the official book giving a verbatim report becomes a permanent record in the library, always accessible. If the proceedings are published in a journal owned and maintained by the society, they become scattered through a whole year, and are often difficult to find when wanted, as the particular number of the journal desired is either mislaid, lost, not received in the mail, or loaned to a friend who has forgotten to return it.

Every experienced worker approves the annual transactions plan of publication, whereas only the theorist favors the medical journal ownership and method.

A TREATISE ON FRACTURES AND DISLOCATIONS. For Practitioners and Students. By LEWIS A. STIMSON, B. A., M. D., Professor of Surgery in Cornell University Medical College, New York. New (3d) edition. In one 8vo volume of 842 pages, with 336 engravings and 32 full-page plates. Philadelphia and New York: Lea Brothers & Co. 1900. [Price, cloth, $5.00 net; leather, $6.00 net.]

Fractures and dislocations occur so often and indeed, constitute such a large proportion of the surgical practice of the average physician and surgeon, that it behooves him to become thoroughly familiar with the subject at the hands of the best teachers and thorough familiarity with the best authors. Stimson ranks as such an one, and his treatise is one of the best and most practical dealing with fractures and dislocations in the English language.

Since the work first appeared in two volumes, for the sake of convenience, it has been reduced to a single volume; this was in the edition of 1899 that became exhausted within a year. The advances in the methods of diagnosis and treatment rendered it necessary to rewrite the fracture section to a large extent for the second edition. In this one all of the improvements are embodied, including notable changes relating to dislocation.

The student will find this treatise par excellence, for his use, and the practitioner cannot fail to appreciate its value for reference. It is well illustrated, especially have the *x*-rays been made to serve an invaluable purpose in the delineation of many bone and joint injuries that heretofore have defied the engraver's art.

Professor Stimson deserves the congratulations of his surgical colleagues, as well as of all his other readers upon the excellent treatise that he has given us.

---

"FESTSCHRIFT" in honor of Abraham Jacobi, M. D., L.L.D., to Commemorate the Seventieth Anniversary of his Birth. May 6, 1900. International contributions to Medical Literature. New York: The Knickerbocker Press. 1900.

Owing to the fact that this volume was misdirected, its delivery to the JOURNAL has not been made apparent until lately, hence the delay in noticing this splendid testimonial to our distinguished confrére. It is vouchsafed to but few men to attain the esteem of his professional colleagues to the degree that Dr. Jacobi has done. This book will testify to that fact as long as time shall wend its way adown the ages.

The particulars that led to this form of testimonial have been so recently published that everyone is familiar with them, hence need not be repeated here. The "Festschrift" contains the contributions of fifty-five men of distinction, residing in this country and in Europe, all written for the special occasion and for this book.

Obviously, it is a volume that cannot be reviewed in the sense of the word as applied to a treatise on practise, but we desire to record our appreciation of the work, not only for the splendid tribute it pays to one of our most distinguished American physicians, but, likewise, for its scientific value. A splendid etching of Dr. Jacobi adorns the festschrift as a frontispiece.

DISEASES OF THE INTESTINES. A Handbook for Practitioners and Students of Medicine. By MAX EINHORN, M. D., Professor of Medicine at the New York Post-Graduate Medical School and Hospital, and Visiting Physician at the German Dispensary, New York. Small 8vo, pp. xiv.—391. New York: William Wood & Co. 1900.

The reputation so justly made by this author through his treatise on diseases of the stomach, issued a few years ago, justifies the expectation that this work will prove of great value to the profession. It is, indeed, a continuation of that subject and, taken together, the two furnish the most complete exposition of the principal diseases of the digestive tract that has yet appeared.

In this book will be found carefully set forth a description of the methods of examination and treatment, which is really the most important subdivision of the treatise. This is a subject less understood by the average physician than any other belonging to the study of intestinal disorders, and will prove of great assistance to every one who has to do with general practice.

Other chapters are devoted to the consideration of special conditions, such as hemorrhoids, appendicitis, intestinal obstruction, nervous affections of the intestines and intestinal parasites, and present these conditions in most excellent form. Especially to be commended is the chapter relating to nervous disorders of the intestines, which deals with obscure conditions heretofore in many respects little understood or greatly neglected. We predict a favorable reception of this treatise by the medical profession in this country.

TRANSACTIONS OF THE AMERICAN SURGICAL ASSOCIATION. Volume XVIII. Session, held May 1-3, 1900. Edited by DEFOREST WILLARD, M. D., Ph.D., Recorder of the Association. Philadelphia; Wm. J. Dornan, Printer.

This volume contains the papers read before this distinguished association, May 1-3, 1900, at which time Dr. Roswell Park, of Buffalo, was elected president. He will preside at the meeting to be held at Baltimore, May 7-9, 1901.

The first paper in this book is the address of the president, Dr. Robert F. Weir, of New York, who chose for his subject Perforating ulcer of the duodenum. This exceedingly rare affection is learnedly discoursed upon, and the author's own experience, limited to a single case, is graphically described, fatal results following an operation. He gives in detail fifty-one cases of the disease, which makes the paper the most complete contribution to the literature of the subject that is in existence. A number of papers follow on subjects akin to this, and the entire volume is replete with excellent contributions to the literature of surgery.

This association always issues a book full of interest and instruction, and this one is no exception to a well-established rule.

---

INTERNATIONAL CLINICS. A Quarterly of Clinical Lectures and Especially Prepared Articles on Medicine, Neurology, Surgery, Therapeutics, Obstetrics, Pediatrics, Pathology, Dermatology, Diseases of the Eye, Ear, Nose and Throat, etc. Edited by HENRY W. CATTELL, A. M., M. D., with the collaboration of John B. Murphy, M. D., of Chicago; Alexander D. Blockader, M. D., of Montreal; H C. Wood, M. D., of Philadelphia; T. M. Rotch, M D., of Boston; E. Landolt, M. D., of Paris; Thomas G. Morton, M. D., and Charles H. Reed, M. D., of Philadelphia. Volume III. Tenth series. 1900. Philadelphia; J. B. Lippincott Co.

The contents of this number embrace papers or lectures on genito-urinary diseases, therapeutics, medicine, neurology, surgery, obstetrics and gynecology, diseases of the eye and ear, laboratory method, and a monograph on the scientific modification of milk.

The foreign contributors are: J. W. Ballantyne, A. Boissard, Merigot de Treiguy, Alfred Fournier, Fernand Lagrange, A. B. Marfau, A. Pinard and Fr. Rubenstein.

Dr. William C. Krauss contributes an instructive article on the use and care of the microscope. Dr. L. Webster Fox discourses upon injuries of the eyelids and eyeballs in a well-illustrated lecture. There are many others of value, indeed, this number is replete with excellent lectures.

---

TRANSACTIONS OF THE TWENTY-FIRST ANNUAL MEETING OF THE AMERICAN LARYNGOLOGICAL ASSOCIATION. Held in the City of Chicago, Ill., May 22, 23 and 24, 1899. New York: D. Appleton & Co. 1900.

At the meeting during which these proceedings were had Dr. William E. Casselbery, of Chicago, served as president. In his address he referred to the fact that the association was organised at Buffalo, June 3, 1878.

Since that time great progress has been made in the field of work occupied by this distinguished body of men. They, themselves, have been the chief promoters of the scientific advance of laryngology in this country, and must view with satisfaction the work they record in these annual volumes. The books though unpretentious show careful editing, and each contains many papers of value, while some are distinguished for originality of thought or for new methods of procedure. Dr. Frank Whitehill Hinkel, of Buffalo, contributes an interesting paper to this volume on the Operative treatment of Frontal and maxillary sinusitis. Other papers of value, numbering twenty-eight in all, serve to make a volume of interest to every laryngologist.

